# Social support for women of reproductive age and its predictors: a population-based study

**DOI:** 10.1186/1472-6874-12-30

**Published:** 2012-09-18

**Authors:** Azam Baheiraei, Mojgan Mirghafourvand, Eesa Mohammadi, Sakineh Mohammad-Alizadeh Charandabi, Saharnaz Nedjat

**Affiliations:** 1 Department of Reproductive Health, Tehran University of Medical Sciences, Tehran, Iran; 2 Centers for Community-Based Participatory Research, Tehran University of Medical Sciences, Tehran, Iran; 3 Department of Midwifery, Tabriz University of Medical Sciences, Tabriz, Iran; 4 Department of Nursing, Tarbiat Modares University, Tehran, Iran; 5 Department of Epidemiology and Biostatistics, Tehran University of Medical Sciences, Tehran, Iran

## Abstract

**Background:**

Social support is an exchange of resources between at least two individuals perceived by the provider or recipient to be intended to promote the health of the recipient. Social support is a major determinant of health. The objective of this study was to determine the perceived social support and its associated sociodemographic factors among women of reproductive age.

**Methods:**

This was a population-based cross-sectional study with multistage random cluster sampling of 1359 women of reproductive age. Data were collected using questionnaires on sociodemographic factors and perceived social support (PRQ85-Part 2). The relationship between the dependent variable (perceived social support) and the independent variables (sociodemographic characteristics) was analyzed using the multivariable linear regression model.

**Results:**

The mean score of social support was 134.3 ± 17.9. Women scored highest in the “worth” dimension and lowest in the “social integration” dimension. Multivariable linear regression analysis indicated that the variables of education, spouse’s occupation, Sufficiency of income for expenses and primary support source were significantly related to the perceived social support.

**Conclusion:**

Sociodemographic factors affect social support and could be considered in planning interventions to improve social support for Iranian women.

## Background

Social support refers to the emotional and material resources that are provided to an individual through interpersonal communications [1]. Social support is an exchange of resources between at least two individuals; resources perceived by the provider or the recipient to be intended to promote the health of the recipient [2,3]. Although numerous definitions have been used to evaluate the concept of social support, it falls into two categories. Objective or received social support indicates what people have actually received or report to have received. The other is a subjective or perceived social support, which captures an individual’s beliefs about the available support [4]. Social support includes three main aspects, each of which may be experienced as positive or negative: emotional (e.g. feeling loved, valued, and appreciated), informational (e.g. advice or guidance), and instrumental (e.g. tangible help) [5].

In human interactions, individuals and groups offer and receive social support. Social support is a mutual process and a source of interaction that provides comfort, assistance, and encouragement. It enhances successful compatibility and improves satisfaction and efficient life [6]. The determinants of perceived social support are primarily divided into four groups: 1) sociodemographic characteristics, which includes age, gender, education, Ethnicity/nativity status, culture and socioeconomic status; 2) social network characteristics such as number of network members and frequency of contact with network members, 3) social integration and involvement characteristics, which refer to the participation of people in a broad range of social relationships, which are measured by marital status, living arrangements, working status, club membership, and religious activities; and 4) health characteristics, which are perceived health status, chronic diseases, and stress [4,7-9].

Social support has been viewed as integral to health promotion as it assists in satisfying an individual's physical and emotional needs, as well as buffering the effects of stressful events on the quality of life [10]. If social support is perceived as helpful, the individual’s health and wellbeing improves, whereas lack of social support increases the risk of disease [11].

Social support is associated with numerous psychological benefits, such as improved self-confidence, sense of empowerment, efficiency, and quality of life. Similarly, lack of social support appears to be related to mental manifestations and weaker health perceptions [12]. Different studies on different populations have indicated positive perception of social support to improve physical health [1,13], health-promoting behaviors [14-19], quality of life [20-22], mental health [23-26], and self-confidence [27], as well as epidemiological studies showed that individuals with low levels of social support have higher mortality rates; especially from cardiovascular disease, cancer, cachexia, and infection-related mortalities [28-30]. These studies have indicated the importance of perceived social support. Wills and Cohen reported that for health-promoting behaviors, perceived social support is more important than actual social support. They pointed out that if resources of support are not perceived by a person, such resources cannot be used [31].

Iranian culture, as a collectivist culture, emphasizes social networks and support [32]. Several studies were conducted in different Iranian populations such as HIV positive-patients [33], students [34], adolescents [35], diabetic patients [36], hemodialysis patients (22), cancer patients [37], Elders [38], and so on, but social support among women of reproductive age has not been investigated in Iran, despite the fact that women of reproductive age constitute a considerable part of the country’s population. In 2006, there were 21 million women of reproductive age (aged 15–49); about 18% of them (3.8 million) lived in Tehran and constituted about 60% of the female population in Iran [39]. The age period of reproduction is associated with a number of stressful events such as pregnancy and lactation; certain disorders, such as depression, anxiety, and nutritional disorders, are more frequent among women [40]. Previous studies have indicated the role of social support in reducing stress and improving health [1,41]. This study aimed to determine the perception of Iranian women of reproductive age of social support and its associated sociodemographic factors.

## Methods

### Study population and data collection

This was a population-based cross-sectional study involving 1359 Iranian women of 15–49 years in Tehran, recruited using multistage cluster sampling. Tehran is divided into 22 municipal districts. Initially, 135 domains were selected using probability sampling weighted with the number of families in each district (proportional to size sampling) and one block was selected at random from each domain. Subsequently, 10 families were selected from each block using systematic sampling. For each family, a woman aged 15–49 answered the question. Thus, 1359 women of reproductive age were selected for the study. All participants were interviewed individually in their homes by a team of interviewers. Response rate for the study population was approximately 90%. If a woman was not at home or was unwilling to participate in the study, the interviewer would refer the next right home and to have the questionnaire completed. Interviewers were trained to administer the questionnaire in a standardized procedure. For quality control of data collection, implementation of this study was completely monitored by the supervisor team.

Informed consent was obtained from all women and the study protocol was approved by the Ethics Committee of the Tehran University of Medical Sciences in Tehran, Iran. The study protocol and eligibility criteria of the participants have been described elsewhere [42]. Each participant was interviewed face to face. Data were collected using questionnaires including sociodemographic characteristics and perceived social support. The sociodemographic factors included age, marital status, education, occupation, sufficiency of income for expenses, crowding index, primary support source, and ethnicity, as well as spouse’s level of education and occupation for married participants. The crowding index was categorized into three levels: low crowding (less than 2 people per room), average crowding (2–3 people per room), and high crowding (more than 3 people per room).

The Personal Resource Questionnaire 85-Part 2 (PRQ85-Part 2) was used to measure the perceived social support. Perceived social support is more persistently and more powerfully related to health and well being than are objective measures. Thus, this study chose to focus on perceived social support, which reflects an individual’s feeling that he/she is accepted, loved, and valued by other members of their social network. The PRQ85-Part 2 was chosen for this study for its ease of use, clarity, and proven reliability and validity in measuring perceived social support. It has been used in health research because of its convenience of use with subjects of differing age groups and ethnicities. Therefore, offers possibilities for comparison across nations and populations. Written consent to use the PRQ85-Part 2 was obtained from Dr. Weinert.

PRQ85-Part 2 is a 25-item scale based on the five dimensions of support, namely worth (the indication that one is valued), social integration (being an integral part of a group), intimacy (provision for attachment/intimacy), nurturance (opportunity for nurturing behavior towards their family and friends), and assistance (the availability of information, emotional, and material help). This questionnaire is based on a 7-point Likert scale ranging from strongly disagree to strongly agree; the score for the questionnaire ranges from 25 to 175 for the entire questionnaire, and from 7 to 35 for its dimensions [43]. In the present study, Cronbach’s α and the intra-class correlation coefficient (ICC) for this tool were 0.84 and 0.9, respectively.

### Data analysis

Sociodemographic characteristics and social support were assessed descriptively with frequency, percentage, mean, standard deviation, median and IQR. The one-way ANOVA test was used to investigate the relationship between perceived social support and sociodemographic characteristics. Backward multiple linear regression analysis was used to predict the impacts of each of the independent variables (sociodemographic characteristics) on the dependent variables (social support) and to determine the variance. Assumptions related to multiple regression including multicollinearity, normality, homoscedasticity, outliers, missing were assessed. Independent variables with p < 0.2 on bivariate test [44] entered the backward regression model. Interaction term analyses were conducted to examine the relationship between predictors of social support and outcome of it. Also, Repeat analyses were conducted for the dimensions of social support. Data were analyzed using SPSS 16.

## Results

### Participants’ characteristics

More than one third of the women were aged 35 years or older, with a mean age of 31.9 ± 9.5 years. Most women were married and housewives. Almost 70% stated that their monthly income sufficed for their expenses. The majority of women (83.8%) identified their spouse or parents as the first persons who provide support for them when they need help. Almost a quarter of the married participants' spouses (28.5%) had a university education, and 43.4% worked in the private sector.

### Perceived social support

The mean score of social support was 134.3 ± 17.9. The highest scored dimension of social support was “an indication that one is valued” whereas the lowest scored dimension was “the feeling of being an integral part of a group” (Table 1).

**Table 1:** Mean and Standard deviation for the social support scale and its dimensions

**Variable**	**Mean (SD)**	**Observed range of score**	**Median**	IQR*
PRQ85-Part 2	134.3 (17.9)	62–175	136	24
Worth	28.3 (4.2)	11–35	29	5
Social integration	24.9 (4.7)	6–35	25	6
Intimacy	25.4 (4.7)	7–35	26	7
Nurturance	27.6 (4.6)	8–35	28	6
Assistance	28.0 (4.6)	7–35	29	7

* Interquartile Range.

### Perceived social support and its relationship with sociodemographic factors

According to the one-way ANOVA test results, there was a statistically significant relationship between perceived social support and education, spouse's education and occupation and sufficiency of income for expenses (*p* < 0.001), primary support source (*p* < 0.01) and occupation (*p* < 0.05) (Table 2). The variables of education, spouse’s occupation, sufficiency of income for expenses, crowding index, primary support source, and ethnicity entered the backward multivariable linear regression model. The variables of crowding index and ethnicity were excluded from the model. The variables of education, spouse’s occupation, sufficiency of income for expenses, and primary support source were significantly related to perceived social support. The results of multivariable linear regression and the significant results of interaction term analyses were showed in Table 3. According to the results of repeat analyses for dimensions of perceived social support, only there was a statistically significant interaction of education * primary source support in the model (Wilks Lambda = 0.95, F (10, 2244) = 1.55, P = O.O4). The results of repeat analyses for dimensions of perceived social support are available as supplementary files (Additional File 1).

**Table 2:** Association between Sociodemographic characteristics and Social support score: Bivariate test (n = 1359)

**Characteristic**^**a**^	**n (%)**	**PRQ-85 Mean ± SD**	**Characteristic**^**a**^	**n (%)**	**PRQ-85 Mean ± SD**
**Age (in years)**			**Spouse’s occupation**^§^		
15–24	350 (25.8)	133.7 ± 18.3	Unemployed	19 (2.0)	127.3 ± 18.7^*^
25–34	468 (34.4)	134.5 ± 17.3	Worker	127 (13.2)	128.9 ± 15.8
35 or higher	541 (39.8)	134.7 ± 18.2	Clerk	363 (37.8)	136.4 ± 17.0
**Marital status**			Private sector	417 (43.4)	134.8 ± 17.7
Single	360 (26.6)	133.3 ± 18.9	Experts/Managers	44 (3.5)	139.6 ± 19.1
Married	957 (70.8)	134.8 ± 17.5	**Sufficiency of income for expenses**		
Divorced	17 (1.3)	133.4 ± 20.7	Absolutely not	199 (14.9)	129.1 ± 20.4^*^
Widowed	17 (1.3)	137.2 ± 13.4	To some extent	928 (69.3)	134.1 ± 17.5
**Education**			Completely	213 (15.9)	140.0 ± 16.0
Illiterate	23 (1.7)	129.1 ± 18.1^*^	Crowding index***		
Elementary school	111 (8.2)	133.7 ± 15.1	Low	611 (46.6)	135.7 ± 18.5
Secondary school	170 (12.5)	132.1 ± 18.5	Average	535 (40.8)	133.2 ± 17.9
High school	86 (6.3)	127.1 ± 17.1	High	164 (12.5)	132.9 ± 15.7
Diploma	510 (37.6)	133.8 ± 17.2	**Primary source support**		
University	455 (33.6)	137.5 ± 18.6	Mother	248 (18.5)	136.0 ± 18.6^**^
**Occupation**			Father	46 (3.4)	131.9 ± 20.2
Housewife	866 (64.1)	133.6 ± 17.7^**^	Mother & Father	214 (16.0)	134.5 ± 17.0
Employed	207 (15.3)	137.9 ± 17.2	Spouse	614 (45.9)	135.3 ± 17.1
Student	247 (18.3)	134.2 ± 18.5	Friend & Relative	188 (14.2)	131.7 ± 16.9
Unemployed	32 (2.4)	132.4 ± 22.7	Others	27 (2.0)	121.4 ± 29.0
**Spouse’s Education**^§^			**Ethnicity**		
Illiterate	8 (0.8)	133.7 ± 12.0^*^	Persian	851 (63.4)	135.2 ± 18.1
Elementary school	107 (10.9)	130.5 ± 18.4	Azeri	380 (28.3)	132.5 ± 17.4
Secondary school	159 (16.1)	132.6 ± 15.4	Kurd	44 (3.3)	131.3 ± 19.6
High school	50 (5.1)	123.3 ± 15.0	Lore	49 (3.7)	136.0 ± 18.3
Diploma	380 (38.6)	135.2 ± 16.6	Guilac	18 (1.3)	137.7 ± 18.8
University	281 (28.5)	138.7 ± 18.4			

^a^All variables, except age, entail unanswered cases.

^§^ This variable applies to married participants.

* *p* <0.001.

** *p* <0.05.

*** Crowding index was determined by dividing the number of family members by the number of rooms, not considering the bathroom.

**Table 3:** Multivariable linear regression analysis for factors associated with Perceived social support

**Variable**	β (95% CI*)	***P-*** **value**
**Education**		
University	Reference	
Illiterate	29.6 (−13.7 to 72.9)	0.18
Elementary school	5.7 (−11.4 to 22.8)	0.51
Secondary school	7.2 (−8.9 to 23.4)	0.38
High school	−8.4 (−29.9 to 13.1)	0.44
Diploma	3.7 (−4.5 to 11.1)	0.37
**Spouse’s occupation**		
Clerk	Reference	
Unemployed	7.1 (−23.7 to 37.9)	0.65
Worker	−30.1 (−58.1 to −2.2)	0.035
Private sector	−6.6 (−14.6 to 1.3)	0.10
Experts/Managers	3.3 (−9.4 to 15.9)	0.61
**Sufficiency of income for expenses**		
Completely	Reference	
To some extent	−12.5 (−24.5 to −0.4)	0.043
Absolutely not	−1.6 (−8.6 to 5.3)	0.65
**Primary source of support**		
Mother, father and spouse	Reference	
Other	−12.8 (−26.8 to 1.7)	0.079
Spouse’s occupation(worker)*Sufficiency of income for expenses (Absolutely not)	29.8 (2.9 to 56.6)	0.030
Primary source of support (other)* education(Illiterate)	27.4 (1.4 to 53.4)	0.038
Primary source of support (other)* education(Elementary school)	15.5 (1.9 to 29.0)	0.025
Primary source of support (other)* education(High school)	26.2 (7.0 to 45.3)	0.007
Primary source of support (other)* education(Diploma)	11.7 (0.2 to 23.2)	0.047
education(Illiterate)* Sufficiency of income for expenses (Absolutely not)	−46.2 (−89.1 to −3.3)	0.035
Education(Illiterate)* Sufficiency of income for expenses (To some extent)	−39.5 (−78.4 to −0.5)	0.047
Education(Diploma)* Sufficiency of income for expenses (To some extent)	−9.2 (−17.3 to −1.1)	0.026
R^2^ = 0.136 Adjusted R^2^ = 0.069		

* CI = Confidence Interval.

## Discussion

In this study, perceived social support was relatively high in Iranian women of reproductive age. Women scored highest in “worth” and lowest in “social integration”. The variables of education, spouse’s occupation, sufficiency of income for expenses, and primary support source were significantly related to perceived social support.

The mean score of perceived social support in the present study was higher compared with those in studies by Adams et al. (2000) on rural women in a southern state of the United States (121.7 ± 14.1) [11], Hovey and Magana (2002) on immigrant farmers in Mexico (132.9 ± 26.5) [23], and Rambod and Rafii (2010) on patients undergoing hemodialysis in Iran (131.9 ± 25.8) [22], whereas it was lower compared with those in studies by Doucette (2000) on patients with congestive heart failure in Canada (141.2 ± 20.9) [45], and Dalla et al. (2006) on paraprofessional educators in the state of Nebraska in the United States (147.4 ± 19.2) [24]. The different results of mean score of social support observed in diverse populations may be due to the effects of a range of sociodemographic, social network, and personality characteristics and social involvement that have been linked to perceptions about social support [7].

Women scored highest in “worth” dimension that is consistent with the results of Doucette (2000) [45]. This dimension explores reassurance of worth as an individual and in role accomplishments. This finding showed that **women** perceived themselves to be secure in their roles and relationships with family and friends as well as revealed that they were comfortable giving and receiving affection, and as individuals, their degree of self-worth was high.

Women scored lowest in “social integration” that is consistent with the results of Doucette (2000) [45] and Kuhirunyaratn et al. (2007) [4]. Social integration is defined as the existence of social ties and typically includes such indicators as: marital status, close family and friends, and degree of participation in group and religious affiliation [45]. Social integration through multiple mechanisms is generally associated with better health outcomes. Integrated individuals are subject to social controls that may promote the adoption of healthful behaviors and prevent risky behaviors. Also social network members may also act as sources of information regarding appropriate medical care. In addition, social networks could influence health of individuals by providing effective support [46,47]

The results of this study showed that subjects to a lesser extent felt involved in outside social activities. The fact that most women in this study were housewives and thus tended to be less involved in social activities outside the home may account for the lower scores of social integration in the present study.

The scores of the different dimensions of perceived social support indicated that, in general, the participants perceived that they are valued and cared for, and in times of need there are supportive companions to help them. Social support serves as a protective factor against the stressful events of life and provides the compatibility skills necessary for coping with stress [48]. Due to the stressful physiological events that occur during the reproductive age, such as pregnancy and delivery, it is essential for women of reproductive age to have social support.

The highest mean of perceived social support was observed in women who considered their income to be sufficient for their expenses, while the lowest score was found among women whose spouses were workers. The relationship between high income and high levels of social support has been indicated in previous studies [1,13,22]. Occupation and income constitute the two major components of socioeconomic status. The size of the social network increases with the improvement of socioeconomic status, as the latter provides sufficient resources for the development and maintenance of relationships in a social network [49].

Higher scores of social support were observed in women who mentioned their spouses or parents as their primary sources of support. This finding is consistent with those of Chen et al. (2007), who studied women in their postpartum period, and reported spouses, mothers, and mother-in-laws as the key social support providers [2]. This finding indicates that the social network of family members constitutes an important aspect of social support for women of reproductive age. This could be because of this fact that culture and our religious believes plays an important role between family members and family is as the most important provider of social support in Iran. The importance of family ties has been emphasized in Islamic countries. Islam considers the family as the foundation of Islamic society. Family in Iran is considered as the primary support system for individuals in times of crisis [22].

### Limitations

There are limitations relevant to the cross-sectional design used in this study. First, the cross-sectional design does not lend itself to causal interpretation; no cause effect relationships can be inferred. Second, the data are collected at one point in time in a cross- sectional research design. It measures what exists today and does not attempt to document changes over time, past or future. Third, this study lacks generalizability beyond the geographic area of the participants. In addition, perceived social support assessment of this study is a subjective evaluation; it relies on the women’ perception, mood and attitude which change over time. Therefore, the measurement depends on the women perception only.

### Implications

The findings of this study provide information about perceived social support of reproductive aged women living in Iran. Because women scored lowest in “social integration”, so women must be encouraged to develop positive social bonds. In addition, we need to understand more about social integration characteristics, and the role of the community integration. Sociodemographic factors must be considered in planning interventions aimed at improving social support for women of reproductive age. For example, Interventions that facilitate an increase in the quality or quantity of their social support should be considered in national programmes for women. Literature supports the idea that social support influences health. As health care expands its view of health beyond the physical into the social realm, health care providers must incorporate these ideas into practice to provide more effective care.

## Conclusion

The findings of the present study indicate that women of reproductive age have relatively high perceived social support. They feel they are respected by others, but they tend not to become involved in social groups. Because social support from the family improves the health of women of reproductive age as well as being present in social groups, women must be encouraged to develop positive social bonds. Sociodemographic factors affect social support and must therefore be considered in planning interventions aimed at improving social support for women of reproductive age.

## Abbreviations

PRQ85-PART2: Personal Resource Questionnaire 85-Part 2; WHO: World Health Organization

## Competing interests

The authors declare that they have no competing interests.

## Authors’ contribution

All the authors participated in its design, coordination, analyses, and interpretation of the results, and drafted the manuscript of the study. MM drafted the first version of the manuscript. AB, EM, MM, and SN revised the manuscript. AB critically reviewed the manuscript. All authors read and approved the final manuscript.

## Pre-publication history

The pre-publication history for this paper can be accessed here:

http://www.biomedcentral.com/1472-6874/12/30/prepub

## Supplementary Material

Additional file 1: Table S1 Multivariable Linear Regression Analysis for Factors Associated with Social Integration Subscale. **Table S2.** Multivariable Linear Regression Analysis for Factors Associated with Nurturance Subscale. **Table S3.** Multivariable Linear Regression Analysis for Factors Associated with Worth Subscale. **Table S4.** Multivariable Linear Regression Analysis for Factors Associated with Assistance Subscale. **Table S5.** Multivariable Linear Regression Analysis for Factors Associated with Intimacy Subscale. Click here for file
