# Supplementary material for: Social support for women of reproductive age and its predictors: a population-based study
Source: BMC Womens Health. 2012 Sep 18;12:30. doi: 10.1186/1472-6874-12-30 (PMC3675417; doi:10.1186/1472-6874-12-30)
Supplement: Additional file 1: Table S1 — Multivariable Linear Regression Analysis for Factors Associated with Social Integration Subscale. Table S2. Multivariable Linear Regression Analysis for Factors Associated with Nurturance Subscale. Table S3. Multivariable Linear Regression Analysis for Factors Associated with Worth Subscale. Table S4. Multivariable Linear Regression Analysis for Factors Associated with Assistance Subscale. Table S5. Multivariable Linear Regression Analysis for Factors Associated with Intimacy Subscale. [file 1472-6874-12-30-S1.doc]

**Table 1**- Multivariable Linear Regression Analysis for Factors Associated with Social Integration Subscale

| Variable | β (95% CI*) | | *p-*value |
| --- | --- | --- | --- |
| **Education** | | | |
| University | Reference | |  |
| Illiterate | 7.4 (-4.5 to 19.3) | | 0.22 |
| Elementary school | -0.8 (-5.5 to 3.9) | | 0.74 |
| Secondary school | -1.6 (-6.0 to 2.9) | | 0.48 |
| High school | -1.9 (-7.8 to 4.0) | | 0.54 |
| Diploma | 1.6 (-0.7 to 3.8) | | 0.17 |
| **Spouse’s occupation** | | | |
| Clerk | Reference | |  |
| Unemployed | -2.1 (-10.6 to -6.3) | | 0.62 |
| Worker | -4.6 (-12.3 to 3.1) | | 0.24 |
| Private sector | 1.4 (-2.1 to 4.9) | | 0.43 |
| Experts/Managers | 0.2 (-2.0 to 2.4) | | 0.86 |
| **Sufficiency of income for expenses** | | | |
| Completely | Reference | |  |
| To some extent | -1.1 (-4.4 to 2.2) | | 0.51 |
| Absolutely not | 0.4 (-1.5 to 2.3) | | 0.65 |
| **Primary source of support** | | | |
| Mother, father and spouse | | Reference |  |
| Other | | -2.0 (-5.9 to 1.9) | 0.31 |
| Education(Diploma)* Sufficiency of income for expenses (To some extent) | | -2.3 (-4.5 to -0.1) | 0.043 |

**Table 2**- Multivariable Linear Regression Analysis for Factors Associated with Nurturance Subscale

| Variable | β (95% CI*) | | *p-*value |
| --- | --- | --- | --- |
| **Education** | | | |
| University | Reference | |  |
| Illiterate | 9.8 (-1.7 to 21.3) | | 0.09 |
| Elementary school | 3.4 (-1.1 to 7.9) | | 0.14 |
| Secondary school | 3.0 (-1.3 to 7.3) | | 0.17 |
| High school | 0.6 (-5.1 to 6.3) | | 0.83 |
| Diploma | 2.1 (-0.08 to 4.2) | | 0.06 |
| **Spouse’s occupation** | | | |
| Clerk | Reference | |  |
| Unemployed | 6.1 (-2.0 to 14.3) | | 0.14 |
| Worker | -9.6 (-17.1 to -2.2) | | 0.01 |
| Private sector | -2.3 (-4.5 to -0.2) | | 0.75 |
| Experts/Managers | 0.5 (-2.8 to 3.9) | | 0.03 |
| **Sufficiency of income for expenses** | | | |
| Completely | Reference | |  |
| To some extent | -3.4 (-6.6 to -0.2) | | 0.04 |
| Absolutely not | 0.05 (-1.8 to 1.9) | | 0.96 |
| **Primary source of support** | | | |
| Mother, father and spouse | | Reference |  |
| Other | | -1.7 (-5.4 to 2.0) | 0.37 |
| Spouse’s occupation(Unemployed) *Primary source of support (other) | | -10.9 (-21.2 to -0.7) | 0.036 |
| Spouse’s occupation(Unemployed) * education(Illiterate) | | -11.6 (-21.7 to -1.6) | 0.024 |
| Spouse’s occupation(Unemployed)* education(Diploma) | | -10.1 (-20.3 to -0.04) | 0.049 |
| Spouse’s occupation(Worker)* education  (Secondary school) | | 4.02 (0.06 to 7.9) | 0.047 |
| Spouse’s occupation(Worker)* Sufficiency of income for expenses (Absolutely not) | | 8.1 (1.0 to 15.3) | 0.025 |
| Spouse’s occupation(Private sector)* Sufficiency of income for expenses (To some extent) | | 2.3 (0.19to 4.4) | 0.033 |
| Education(Illiterate)* Sufficiency of income for expenses (To some extent) | | -11.3 (-21.7 to -0.9) | 0.032 |
| Education(Diploma)* Sufficiency of income for expenses (To some extent) | | -2.5 (-4.7 to -0.4) | 0.021 |

**Table 3**- Multivariable Linear Regression Analysis for Factors Associated with Worth Subscale

| Variable | β (95% CI*) | | *p-*value |
| --- | --- | --- | --- |
| **Education** | | | |
| University | Reference | |  |
| Illiterate | -0.5 (-11.0 to 9.9) | | 0.92 |
| Elementary school | -0.2 (-4.3 to 3.9) | | 0.91 |
| Secondary school | 0.3 (-3.6 to 4.2) | | 0.87 |
| High school | -1.5 (-6.7 to 3.7) | | 0.57 |
| Diploma | 0.3 (-1.7 to 2.3) | | 0.77 |
| **Spouse’s occupation** | | | |
| Clerk | Reference | |  |
| Unemployed | 0.5 (-6.9 to 7.9) | | 0.89 |
| Worker | -4.7 (-11.4 to 2.1) | | 0.18 |
| Private sector | -0.8 (-2.8 to 1.1) | | 0.40 |
| Experts/Managers | 1.9 (-1.1 to 5.0) | | 0.21 |
| **Sufficiency of income for expenses** | | | |
| Completely | Reference | |  |
| To some extent | -1.8 (-4.7 to 1.1) | | 0.23 |
| Absolutely not | -0.2 (-1.9 to 1.5) | | 0.82 |
| **Primary source of support** | | | |
| Mother, father and spouse | | Reference |  |
| Other | | -0.5 (-3.9 to 2.9) | 0.79 |
| Primary source of support (other)* education(Elementary school) | | 3.5 (0.23 to 6.8) | 0.036 |
| Primary source of support (other)* education(Secondary school) | | 3.2 (0.04 to 6.4) | 0.047 |
| Primary source of support (other)* education(High school) | | 6.4 (1.8 to 11.0) | 0.007 |

**Table 4**- Multivariable Linear Regression Analysis for Factors Associated with Assistance Subscale

| Variable | β (95% CI*) | *p-*value |
| --- | --- | --- |
| **Education** | | |
| University | Reference |  |
| Illiterate | 5.4 (-5.9 to 16.7) | 0.35 |
| Elementary school | 2.8 (-1.6 to 7.3) | 0.21 |
| Secondary school | 3.7 (-0.5 to 7.9) | 0.08 |
| High school | -2.3 (-7.9 to 3.3) | 0.42 |
| Diploma | 0.2 (-1.9 to 2.4) | 0.81 |
| **Spouse’s occupation** | | |
| Clerk | Reference |  |
| Unemployed | 4.4 (-3.6 to 12.5) | 0.28 |
| Worker | -2.7 (-10.1 to 4.5) | 0.46 |
| Private sector | -0.7 (-2.8 to 1.3) | 0.22 |
| Experts/Managers | 2.1 (-1.2 to 5.4) | 0.48 |
| **Sufficiency of income for expenses** | | |
| Completely | Reference |  |
| To some extent | -1.8 (-4.9 to 1.4) | 0.27 |
| Absolutely not | -0.3 (-2.1 to 1.5) | 0.76 |
| **Primary source of support** | | |
| Mother, father and spouse | Reference |  |
| Other | -6.5 (-10.2 to -2.9) | 0.001 |
| Spouse’s occupation(Unemployment)* Primary source of support (other) | -11.4 (-2.5 to -1.3) | 0.027 |
| Spouse’s occupation(Unemployment)* education(Secondary school) | -10.7 (-20.7 to -0.8) | 0.034 |
| Spouse’s occupation(Unemployment)* education(Diploma) | -10.4 (-20.3 to -0.4) | 0.041 |
| Spouse’s occupation(Private sector)* education(Secondary education) | -2.6 (-5.0 to -0.2) | 0.031 |
| Spouse’s occupation(Unemployment)* Sufficiency of income for expenses (Absolutely not) | 9.11 (1.0 to 17.2) | 0.027 |
| Spouse’s occupation(Private sector)* Sufficiency of income for expenses (Absolutely not) | 2.9 (0.09 to 5.7) | 0.043 |
| Primary source of support (other)* Education(Illiterate) | 14.3 (7.5 to 21.0) | <0.001 |
| Primary source of support (other)* Education(Elementary school) | 3.8 (0.3 to 7.4) | 0.034 |
| Primary source of support (other)* Education(Secondary school) | 3.9 (0.5 to 7.3) | 0.025 |
| Primary source of support (other)* Education(High school) | 5.9 (0.9 to 10.9) | 0.020 |
| Primary source of support (other)* Education(Diploma) | 3.8 (0.8 to 6.9) | 0.012 |
| Education(Secondary school)* Sufficiency of income for expenses (To some extent) | -4.2 (-8.2 to -0.1) | 0.040 |

**Table 5**- Multivariable Linear Regression Analysis for Factors Associated with Intimacy Subscale

| Variable | β (95% CI*) | | *p-*value |  |
| --- | --- | --- | --- | --- |
| **Education** | | | |  |
| University | Reference | |  |  |
| Illiterate | 7.5 (-4.0 to 19.1) | | 0.20 |  |
| Elementary school | 0.49 (-4.1 to 5.0) | | 0.83 |  |
| Secondary school | 1.7 (-2.6 to -6.1) | | 0.43 |  |
| High school | -3.4 (-9.1 to -2.4) | | 0.25 |  |
| Diploma | -0.5 (-2.7 to 1.7) | | 0.64 |  |
| **Spouse’s occupation** | | | |  |
| Clerk | Reference | |  |  |
| Unemployed | -1.8 (-10.0 to -6.4) | | 0.67 |  |
| Worker | -8.5 (-15.9 to -1.0) | | 0.02 |  |
| Private sector | -2.9 (-5.0 to -0.8) | | 0.007 |  |
| Experts/Managers | -2.7 (-6.1 to 0.7) | | 0.12 |  |
| **Sufficiency of income for expenses** | | | |  |
| Completely | Reference | |  |  |
| To some extent | -4.4 (-7.6 to -1.1) | | 0.008 |  |
| Absolutely not | -1.6 (-3.5 to -0.2) | | 0.087 |  |
| **Primary source of support** | | | |  |
| Mother, father and spouse | | Reference |  |  |
| Other | | -1.9 (-5.7 to 1.8) | 0.31 |  |
| Spouse’s occupation( Experts/Managers)* education(Diploma) | | 7.3 (2.7 to 12.0) | 0.002 |  |
| Spouse’s occupation (Private sector)* Sufficiency of income for expenses (Absolutely not) | | 3.4 (0.5 to 6.3) | 0.020 |  |
| Spouse’s occupation (Private sector)* Sufficiency of income for expenses ( To some extent ) | | 2.5 (0.4 to 4.6) | 0.021 |  |
|  | | | | |
